# Contributions to the History of Development of the Teeth

**Published:** 1888-02

**Authors:** Carl Heitzmann, C. F. W. Bödecker


### Independent Practitioner.

Vol. IX. February, 1888. No. 2.

Note.—No paper published or to be published in another journal will be accepted for this department. All papers must be in the hands of the Editor before the first day of the month preceding that in which they are expected to appear. Extra copies will be furnished to each contributor of an accepted original article, and reprints, in pamphlet form, may be had at the cost of the paper, press-work and binding, if ordered when the manuscript is forwarded. The Editor and Publishers are not responsible for the opinions expressed by contributors. The journal is issued promptly. on the first day of each month.

### Original Communications.

### CONTRIBUTIONS TO THE HISTORY OF DEVELOPMENT OF THE TEETH.

BY CARL HEITZMANN, M. D., AND C. F. W. BÖDECKER, D. D. S., M. D. S.

Continued From Page 5.

### VI. LITERATURE.

The literature of the development of the teeth is so extensive that the writers have found it almost impossible to give full abstracts from every author. Some of the works, too, were not at our immediate disposal, and for this reason we have been unable to preserve the proper succession or order of publication.

In the Natural History of the Human Teeth, London, 1778, John Hunter noticed the dental sacs. He also mentions that the incisors and cuspids begin to ossify on their edges about the sixth or seventh month of uterine development.

Delabare, in 1805 (System Handbuch der Zahnheilk. Von G. Carabelli, Wien, 1844, page 139), says that he has observed small openings upon the rim of the jaws, which represent the inlets to the dental sacs. He regards the enamel as a living substance receiving blood-vessels and provided with sensibility.

In the Natural History and Diseases of the Teeth, by Joseph Fox, London, 1814, we find an account of the formation of teeth on page 1, as follows: "As soon as the ossific deposit commences in the cartilaginous parts of the embryo, both jaws are filled with small membranous sacs. When the gum which covers the alveolar groove of a fœtus of four months' old is stripped off from the bone, small processes or elongations from the inner surface of the gums may be directly perceived. These are the first appearances of the pulps from which the teeth are formed." Fox also observed that the tooth-sacs about the fourth fœtal month were very soft and represented the shape of the tooth. He also observed that ossification of the tips of the incisors and cuspids, as well as the points of molars, commences about the fifth or sixth month, and gradually extends itself over the whole surface down to the neck of the tooth.

One of the first writers who gave definite information concerning the development of the teeth was Fr. Arnold (Medicin. Chirurg. Zeitung. Insbruck, 1831, Bd. II., page 236), who was the first to observe that the tooth sacs (papillæ) were derivations from the oral mucous membrane. He also mentions a peculiar occurrence in the mouth of a new-born infant, which seems to be identical with what the writers have described and depicted in Fig. 35. He says, "Once I observed in the mouth of a new-born infant, behind the protruding ridge of the gums, several openings which led to the sacs of the front and eye-teeth, and which usually are found closed up by the mucous membrane before birth."

These assertions were criticised by Purkinje and Raschkow in 1835, who maintained the opinion that the dental follicles were formed independently of the mucous membrane of the gums. Raschkow, in a foot-note appended to his researches, remarks that he has observed that the enamel organ receives blood-vessels in certain parts, and believes the parenchyma of the organ to be pervaded by capillary vessels. The conclusion which he deduces from this observation is that the enamel organ was from the beginning joined to the capsule. (Nasmyth, page 109.)

Thomas Bell (The Anatomy, Physiology and Diseases of the Teeth, Philadelphia, 1837, page 53) observed in embryos about two months old, an extremely soft, jelly-like substance, lying along the edge of each maxillary arch, which in the third month he found of firmer consistency, and contained in a shallow groove, and further on says: "These pulps are the rudiments or bases upon which the teeth are formed, and each is partially enclosed in a membranous sac." At the fourth month, if the sac be opened, a small point of ossification is found to have been deposited, and this is the commencement of the formation of the bony substance of the teeth. The ossific matter is secreted, not from the pulp itself, but from an extremely delicate, thin, vascular membrane, which covers its surface, and is closely attached to it by vessels." He also noticed that the sac consisted of two lamellæ.

Of the formation of the enamel, Bell says: "At this time a remarkable alteration takes place in the substance of the sac, which becomes thickened, and much more vascular. * * * It now begins to pour out from its internal surface a thickish fluid, which is speedily consolidated into a dark, chalky substance, and afterwards becomes white and hardened by more perfect crystallization. This is the enamel."

A. Nasmyth (Researches on the Development, Structure and Diseases of Teeth, London, 1839) endorsed the statements of Arnold and Goodsir. He also gives a description of the enamel organ, describing it as being composed of a variety of cells as to size and shape, and also notices the minute granules in the interstices of the cells, (basis substance of the stellate reticulum). He believed that all the cells of the enamel organ were of the same nature, and regards the narrow and oblong cells, which lie upon the dentine papilla, as in a state of preparation for the reception of the calcareous salts. These cells he believes to have arisen from the oval ones, and those from the flat triangular cells. He also mentions the external epithelial cells of the enamel organ.

He describes the Nasmyth membrane to be the remains of the dental capsule, and to be continuous with the cementum, on the outside of the fang of the tooth, "which latter is itself continued into the chamber of the tooth." (Page 114.)

Richard Owen (Odontography, London, 1840-1845) observed that the matrix of the mammalian teeth sinks into a furrow and becomes enclosed in a cell in the substance of the jaw-bone. The cement he regards as the result of ossification of the tooth capsule. He also mentions that the permanent teeth are derived from the germs of their predecessors. On the formation of dentine, he mentions that the cells on the surface of the pulp are more numerous, and that they are arranged in lines.

Ph. Fr. Blandin, of Paris (Anatomy of the Dental System, Baltimore, 1845, page 38), draws attention to the dental follicle, and compares it with the follicles of the hair and feathers. He describes them as little sacs which are formed by depressions of the mucous membrane. He further says: "The dental organs are essentially composed of two elements, the secreting portion, and the portion secreted." The former includes the matrix follicle and the bulb or germ, and the latter portions of the tooth itself.

Sir John Tomes (A Course of Lectures on Dental Physiology and Surgery, London, 1848), to whom the profession is greatly indebted for his accurate scientific observation in this, as in many other subjects (Lecture 4, page 68), quotes the observations of Goodsir, describing the formation of the primitive dental groove, at the bottom of which a papilla is formed, and then, as he expresses it, the walls of the groove send out lamellæ toward each other, which unite, and by these means the papillæ are enclosed in follicles. He then mentions the different changes of shape the papillæ undergo from the club-shaped enlargement to the perfect enclosure of the tooth. About the fourteenth week Tomes says the primitive dental groove disappears and is succeeded by another groove, which, however, is situated on a little higher level, and is destined to furnish the papillæ of the ten anterior permanent teeth.

In the next lecture (V, page 82) he carefully considers the development of enamel, dentine and cementum, and after a brief mention of the cell theory, as accepted at that early time, he begins to consider the development of the dentine, and says that previous to its formation the inner surface of the sac (enamel organ) becomes separated from the surface of the pulp, the intervening space being occupied by a soft, gelatinous, granular matter, the formative pulp for the development of the enamel. He further says that at that time we find two formative pulps, one for the dentine, the other for that of the enamel in one tooth sac. For convenience sake he gives the development of dentine in three stages. The first he calls the alveolar, the second the cellular, and the third the linear stage, when the cells arrange themselves in rows and become what has later been termed odontoblasts. In regard to the process of calcification, he says: "Each cell after falling into line divides into two or more in its length, and each division elongates. A central nucleus or space is seen in each cell, which lengthens with the cell. The cells, by their increased length, become placed end to end, and ultimately unite, and the elongated central space of each individual, by a further development, joins and opens into those of the super-imposed cells. In chapter II, "On the Development of Dentine," the writers have described (see Fig. 15, C. D.) close to the nearly calcified dentine a light zone composed of non calcified basis-substance of dentine, which seems to have been observed by Tomes at that early time, for on this subject he says: "Covering the pulp is a transparent membrane closely united to the external cells. This membrane, which forms the exterior of the dentine, is the first to undergo calcification." He regards the basis-substance of the dentine identical with that of bone.

On the development of enamel Tomes says that he observed minute blood-vessels freely traversing the enamel-pulp in its first or reticular stage, but the writers have only met with blood-vessels after the breaking down of the external epithelium (see Figs. 5 and 6), in which stage Tomes did not notice them. He then describes the stellate reticulum and the stratum intermedium, and remarks: "The two tissues described graduate into each other, the reticular forming at an early period nineteen-twentieths of the whole mass, and, I believe, originally, all of it. With the gradual advancement of development it disappears, leaving the stellate tissue, and this too, at last, yields its place to a columnar tissue—the enamel matrix. * * * Hence we have existing at one time three formative tissues; the first to be transformed into or give place to the second; and the second into the third, and that into enamel." Concerning the function of the stellate tissues he is of the opinion that they prepare the nutrient fluid for the peculiar wants of the columnar tissue and the development of the enamel. Of the process of calcification, Tomes speaks as follows: "The enamel cells being formed in line, eventually become confluent. * * * The nuclei, from the first, very small, are altogether lost in the formation of the fibers, or exist as very fine tubes passing through the length of each. The lateral union is at this time very slight. * * * The enamel continues for a considerable time to increase in density. * * * Recently developed enamel, from the innumerable interspaces between the fibers, is very opaque or pearly. The interspaces, however, become gradually less numerous, and at last, in perfectly formed enamel, they are almost entirely lost. Thus we have what may be termed a progressive growth towards the perfecting of the tissue of the enamel after the appearance of the tooth through the gum—a fact which has, I think, been overlooked, especially by those who have considered that the dental tissues are devoid of vitality." Of the formation of cementum he mentions that its formative organ is the capsule (sac) of the tooth, but believes that the cementum is formed by a direct process of calcification, a theory at that time universally admitted. He says (page 105): "The nuclei (of the cells) being transparent, present the appearance of cavities, which, indeed, they ultimately become. The parietes of the cells and the interposed granular tissue receive the phosphate and other salts of lime."

A. Kölliker (Entwicklungs-Geschichte des Menschen und der Höheren Thiere, Leipzig, 1879), in describing the enamel organ, says that it belongs entirely to the epithelial tissues, and observed the two layers—the internal and external—epithelium of the enamel organ. He says that the stellate reticulum, in appearance, is identical with connective tissue, but is really nothing but a peculiarly transformed epithelium. He also mentions the stratum intermedium, which, in the first stages of development, is transformed into the tissue of the stellate reticulum. He further says, that between the epithelial elements (ameloblasts), and the papilla is situated a very delicate membrane—membrana præformativa—which has a specific function. He also describes the transformation of the external epithelium into epithelial stems (Epithelialfortsaetze), between which blood-vessels originate, but at the same time states that they never enter the enamel organ. He describes the growing of connective tissue into the lower part of the enamel organ which forms the dentine papilla as well as the formation of the tooth-sac. He also observed the stratification in the enamel, which he attributes to the different periods of its calcification.

W. Waldeyer (Manual of Histology, by S. Stricker, New York, 1872), in his description of the development of teeth (page 339), after mentioning the formation of the primitive groove, describes the enamel organ originating from the oral mucous membrane, at first appearing like a short tubular gland. He explains the further development as follows: "The spheroidal cells forming the central part of the enamel germ begin to increase with rapidity, so that the germ * * * assumes the form of a club." He then describes the manner in which the club grows downward till it has attained the shape of a cup, which envelops the dentine papilla. He mentions the disappearance of the epithelial cord at a later period, but does not state what becomes of it. He observed that the enamel cells (ameloblasts), which rest upon the dentine, with their extremities, become elongated. He also noticed the external and internal epithelial layer, and between these the stratum intermedium, which latter gives rise to the stellate reticulum. On this subject he says further: "The cells lying in immediate contact with the epithelium (stratum intermedium, Hannover), retain their original form, and from these a continuous development of enamel cells, as well as of gelatinous epithelial tissue, appears to proceed." Waldeyer is of opinion that the basis-substance of the enamel organ only serves a mechanical purpose, and further states that before the formation of the enamel is completed, both the stratum intermedium as well as the epithelial and gelatinous tissue atrophy. Nasmyth membrane he believes to be an epithelial formation, which is derived from the external epithelium of the enamel organ. In reference to the calcification of enamel, he believes that the enamel prisms are the result of a direct calcification of the enamel cells (ameloblasts), basing his assertions upon the fact that some of these cells, when detached from young enamel, exhibited the Tomes process. He believes that the light zone, described by Kölliker and Huxley as the membrana præformativa, is nothing but newly-formed enamel, and is detachable, an artificial product. He observed the stratification of the enamel, and described the upward growth of the tooth-sac, which latter furnishes the cementum of the root of the tooth. The process of ossification of the dentine he considers identical with that of ordinary bone. In regard to the formation of dentine, he entertains the view that the dentinal fibers are the central remains of the odontoblasts, while their peripheral portion becomes basis-substance. He also observed that the odontoblasts were connected with each other by fine processes, and believes that each dentinal fiber is formed by a coalescence of several odontoblasts.

One of the best descriptions of the development of teeth, as well as a very complete summary of the literature on this subject, is that of G. Hertz (Virchow's Archiv, 1866, Bd. 37, Heft 3, page 272). He gives a careful description of the formation of the primitive furrow and epithelial hill (Zahnwall), after which he describes the development of the tooth-sacs as well as that of the papillæ. He attributes the club or cup-shaped enlargement of the enamel organ to the upward growth of the dentinal papilla. In regard to the formation of the permanent teeth, Hertz is of the opinion that their germs originate either from the neck of the epithelial cord near the oral mucosa, or directly and separately from the latter at a place near the primitive fold of the temporary tooth. Hertz also gives a good description of the stratification and discoloration of the enamel, which was first noticed by Schreger and Retzius, and later by Czermak, Kölliker and others. Hertz is of the opinion that the origin of this peculiar stratification and pigmentation of the enamel must lay in the altered condition of the enamel prisms. Such enamel, when treated with acids, would leave an organic residue which did not possess the clear aspect that he usually observed in other (healthy) enamel. The residue from the former presented fine, dark granules, which Frank Abbott has announced to be enlarged and beaded enamel fibers.

Hertz then, after giving the different views of Todd, Bowman, Hannover, Purkinje, Raschkow, Schwann, Huxley, Lent, Kölliker, Waldeyer and John Tomes, in regard to the formation of enamel, says, "After careful consideration of all (the work quoted above), I endorse the direct transformation of the enamel cells into enamel prisms." But he says on the next page (294) that he cannot deny, that in young forming enamel the transition from the enamel cells into enamel is apparently not a direct one, as he observed, especially on specimens treated with chromic acid, a small light zone which, perhaps, may appear like a membrane which was situated between the formed enamel and the enamel cells.

In the process of calcification he agrees with Tomes, that first the periphery and then the center of the ameloblasts become impregnated with lime-salts. His views in regard to the stratum intermedium are that this layer is the matrix of the enamel organ for the benefit of the enamel cells (ameloblasts), and says: "By the formation of the stellate reticulum its development is stopped only for the time being, as this tissue and in this condition only serves as a nutritive substance, at least in the parts situated near the internal epithelium, for the formation of the enamel cells and enamel prisms, and later, through a new formation of cells, it contributes directly to the formation of the enamel. In this way the whole stellate reticulum is transformed into the stratum intermedium, and when the stellate reticulum has been used up, the formation of the enamel is completed." Hertz is of opinion that Nasmyth membrane is developed from the remains of the external epithelium. He also observed the protoplasmic bodies situated in layers (interzonal) between the dentine and enamel.

Of the development of dentine he gives (page 314) an excellent grouping of the views of the different authors as follows :—

1. "The basis-substance originates from fibers which are formed by the (dentine) pulp. The dentinal canals represent the spaces between these fibers."—(Raschkow.)

2. "The lengthened and obliterated nuclei of the superficial layer of the pulp form the walls of the dentinal canaliculi, in the surrounding of which the basis-substance is formed, either by the cells alone, or by them together with the intercellular substance."—(Henle, Owen, Hannover, Tomes.)

3. "The basis-substance originates from the cylindrical cells, which blend and ossify together, and the dentinal canaliculi are the remains of the cell cavities."—(Zellenhöhlen, Kölliker.)

4. "The cells form the dentine canaliculi in such a manner that their processes are transformed into the canaliculi around the neighborhood of which the lime-salts are deposited. This theory was first advanced by Schwann, but afterward abandoned, and later again endorsed by Lent."

5. "The dentine cells and their processes represent the dentine fibers. The dentinal canaliculi have no walls. The basis-substance is a secretion of the dentine cells, or the tooth-pulp."—(Kölliker.)

6. "The majority of the protoplasm of the dentinal cells is transformed into a calcified connective tissue basis-substance, surrounding the dentinal canaliculi, a small portion of the protoplasm remaining soft and unaltered as dentine fibers."—(Waldeyer.)

"The last (Waldeyer's) assertion appears at first the most explicable, but after all that I have seen, I have to endorse the opinions of Kölliker, which have been published lately, and which, on page 316, he (Hertz) states to be as follows: 'The dentinal fiber originates directly from the odontoblasts in such a manner that the peripheral portion of the odontoblasts forms the outer wall of the dentinal fibers, while the protoplasm of the cell forms the central portion of the fibers. The basis-substance is the chemically altered and calcified intercellular substance of the odontoblasts in which the dentine canaliculi are present as channels without walls.' "

On the development of the cementum this author says nothing but that his researches have not been completed.

(TO BE CONTINUED.)

